# Selected maternal morbidities in women with a prior caesarean delivery planning vaginal birth or elective repeat caesarean section: a retrospective cohort analysis using data from the UK Obstetric Surveillance System

**DOI:** 10.1136/bmjopen-2014-007434

**Published:** 2015-06-02

**Authors:** Manisha Nair, Kate Soffer, Nudrat Noor, Marian Knight, Malcolm Griffiths

**Affiliations:** 1 National Perinatal Epidemiology Unit (NPEU), Nuffield Department of Population Health, University of Oxford, Oxford, UK; 2 Department of Obstetrics & Gynaecology, Luton and Dunstable University Hospital, Luton, UK

**Keywords:** Vaginal Birth after Caesarean, Elective Repeat Caesarean Section, Peripartum hysterectomy, Severe sepsis, Peripartum haemorrhage, Failed tracheal intubation

## Abstract

**Objective:**

To conduct a secondary analysis of data from the UK Obstetric Surveillance System (UKOSS) to estimate the rates of specific maternal risks associated with planned vaginal birth after caesarean (VBAC) and elective repeat caesarean section (ERCS).

**Design:**

A retrospective cohort analysis using UKOSS data from 4 studies conducted between 2005 and 2012.

**Setting:**

All hospitals with consultant-led maternity units in the UK.

**Population:**

Pregnant women who had a previous caesarean section.

**Method:**

Women who had undergone a previous caesarean section were divided into 2 exposure groups: planned VBAC and ERCS. We calculated the incidence of each of the 4 outcomes of interest with 95% CIs for the 2 exposure groups using proxy denominators (total estimated VBAC and ERCS maternities in a given year). Incidences were compared between groups using χ^2^ test or Fisher's exact test and risk ratios with 95% CI.

**Main outcome measures:**

Severe maternal morbidities: peripartum hysterectomy, severe sepsis, peripartum haemorrhage and failed tracheal intubation.

**Results:**

The risks of all complications examined in both groups were low. The rates of peripartum hysterectomy, severe sepsis, peripartum haemorrhage and failed tracheal intubation were not significantly different between the 2 groups in absolute or relative terms.

**Conclusions:**

While the risk of uterine rupture in the VBAC and ERCS groups is well understood, this national study did not demonstrate any other clear differences in the outcomes we examined. The absolute and relative risks of maternal complications were small in both groups. Large epidemiological studies could further help to assess whether the incidence of these rare outcomes would significantly differ between the VBAC and ERCS groups if a larger number of cases were to be examined. In the interim, this study provides important information to help pregnant women in their decision-making process.

**Strengths and limitations of this study**

- While the risk of uterine rupture associated with vaginal birth after caesarean (VBAC) is known, this study estimated the rates of other specific maternal risks (peripartum hysterectomy, severe sepsis, peripartum haemorrhage and failed tracheal intubation) associated with VBAC and elective repeat caesarean section (ERCS) using existing national data from the UK Obstetrics Surveillance System (UKOSS).
- The low incidence of severe maternal morbidities in the UK makes it difficult to compare the risks between the VBAC and ERCS groups. The UKOSS database of research data on rare and potentially life-threatening conditions in pregnancy provided a unique opportunity to estimate the risk of the four adverse maternal outcomes between the two groups in a cost-effective manner.
- The method used to generate the exposure groups (planned VBAC and ERCS) could have misclassified some women who were planning ERCS, but went into spontaneous labour and were thus included under the VBAC group. However, we do not anticipate a large proportion of such women.
- Cases which could not be grouped into VBAC or ERCS due to missing information could have biased the study results, mainly for the sepsis group. We have thus reported the results of a sensitivity analysis.
- A large epidemiological study with a greater number of cases would improve the power and possibly show significant differences in the outcomes; however, this study intended to take advantage of existing secondary data, and the results could pave the way for further studies.

## Introduction

Current UK guidelines^1^ ^2^ advise that women who have undergone a prior delivery by caesarean section should be informed of the risks and benefits of elective repeat caesarean section (ERCS) as well as the risks and benefits of planned vaginal birth after caesarean (VBAC). Such a discussion requires comprehensive evidence of the risks associated with ERCS compared with VBAC. Several studies have examined the risk of uterine rupture following VBAC,^3–5^ but robust data comparing a wider range of complications of VBAC and ERCS are limited, and the few randomised controlled studies^6^ ^7^ have limitations.

A previous study in the UK demonstrated uterine rupture to be associated with VBAC.^8^ Uterine rupture is a rare and serious complication of VBAC, but when comparing ERCS and VBAC it is important to consider other maternal complications. The aim of this study was therefore to estimate the rates of other specific maternal risks associated with VBAC and ERCS using available national data from the UK Obstetric Surveillance System (UKOSS).

## Methods

### Study design

We conducted a retrospective cohort analysis using data from the UKOSS. Details of the UKOSS methodology are described elsewhere.^9^ ^10^ UKOSS was set up in 2005 to investigate uncommon disorders of pregnancy and ‘near-miss’ conditions.^10^ Case notification cards are sent to all consultant-led obstetric units in the UK every month. An approach of ‘nil-reporting’ together with a rigorous follow-up of non-responders ensures good case ascertainment. For every case reported, details are completed in a data collection form by the clinician responsible for managing the case.

Exposure groups were planned VBAC and ERCS. Women who had a history of caesarean section and underwent elective caesarean section during their current pregnancy were included in the ERCS group. Women who had a previous caesarean section but planned vaginal delivery during the current pregnancy were included in the planned VBAC group irrespective of whether they actually had a vaginal delivery.

Outcomes of interest were maternal complications like peripartum hysterectomy, severe sepsis, peripartum haemorrhage and failed tracheal intubation, which are suggested to be related to VBAC or ERCS in other studies.^6^ ^11^ ^12^ We had national data sets within UKOSS for the outcomes (peripartum hysterectomy,^13^ severe sepsis,^14^ peripartum haemorrhage^15^ ^16^ and failed tracheal intubation^17^), and thus case definitions were based on the standard case definitions used in the UKOSS (provided in table 1).

**Table 1:** Definitions of outcomes included from the UKOSS national studies

Condition	Definition
Peripartum hysterectomy	Any woman giving birth to an infant and having a hysterectomy during the same clinical episode
Peripartum haemorrhage	Cases were pregnant women of 20 weeks gestation or more identified as having >8 units of red blood cell transfusion within a 24 h period
Failed tracheal intubation	A case of failed intubation was defined as failure to achieve tracheal intubation during a rapid sequence induction for obstetric anaesthesia, thereby initiating a failed intubation drill
Severe sepsis	Any pregnant woman (up to 6 weeks postpartum) diagnosed with severe sepsis (irrespective of the source of infection). A severe sepsis case would be expected to include women in one of the following groups: Death related to infection or suspected infection Any women requiring level 2 or level 3 critical care (or obstetric HDU-type care) due to severe sepsis or suspected severe sepsis A clinical diagnosis of severe sepsis—based on two or more of the following: Temperature >38°C or <36°C measured on two occasions at least 4 h apart Heart rate >100 bpm measured on two occasions at least 4 h apart Respiratory rate >20/min measured on two occasions at least 4 h apart White cell count >17×10^9^/L or <4×10^9^/L or with >10% immature band forms, measured on two occasions

UKOSS, UK Obstetric Surveillance System; HDU, high dependency unit.

### Study sample

**Figure 1:**
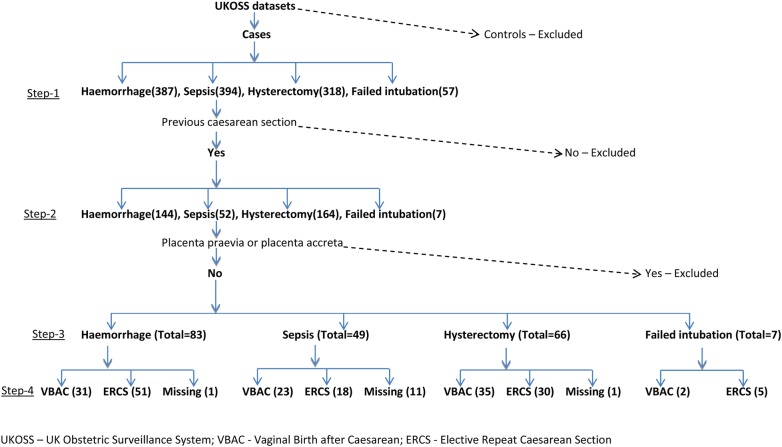
Schematic diagram of derivation of study sample.

For each of the four maternal outcomes for which a national data set was available, we used the total reported cases. The data sets were from four different UKOSS studies; thus, the data included were from different time periods corresponding to the data collection period for each study (table 2). Among the cases, those without a previous history of caesarean section were excluded. We also excluded women with placenta praevia/accreta/percreta diagnosed before delivery to exclude known confounding due to these conditions, which would be regarded as an absolute indication for ERCS. The final sample of cases that remained were women with any previous caesarean sections, and these were further divided into the planned VBAC and ERCS groups on the basis of the planned mode of delivery. If a data set did not include information on the ‘planned mode of delivery’, we investigated two other variables: ‘woman underwent induction of labour with or without prostaglandins and/or oxytocin’ and ‘woman went into labour’. If either of these was ‘true’, we categorised the woman as planned VBAC (irrespective of her actual mode of delivery—vaginal or caesarean), otherwise as ERCS. If information on any of these criteria was not available, we grouped the cases into a missing category. A schematic diagram of the process of derivation of the study samples for peripartum hysterectomy, severe sepsis, peripartum haemorrhage and failed intubation is provided in figure 1.

**Table 2:** Rate of severe maternal morbidities in the VBAC and ERCS groups

Conditions	Study period	Total maternities for the study period	Maternities with previous Caesarean deliveries*	VBAC			ERCS			p Value of χ^2^ test§ for outcome difference between VBAC and ERCS	Risk ratios (VBAC to ERCS) (95% CI)
				Maternities with previous CS with VBAC^†^	Cases	Estimated rate per 100 000 maternities (95% CI)	Maternities with previous CS with ERCS‡	Cases	Estimated rate per 100 000 maternities (95% CI)		
Uterine rupture^8^	01/04/2009 to 30/04/2010	852 206	127 831	56 246	116	206.7 (170.5 to 247.3)	71 585	20	27.9 (17.1 to 43.2)	<0.001	7.39 (4.58 to 12.55)
Peripartum hysterectomy	01/02/2005 to 29/02/2006	839 785	109 172	48 036	35	72.9 (50.8 to 101.3)	61 136	30	49.1 (33.1 to 70.0)	0.110	1.49 (0.89 to 2.50)
Sepsis	01/06/2011 to 31/05/2012	801 770	104 231	45 861	23	50.1 (31.7 to 75.2)	58 370	18	30.8 (18.3 to 48.7)	0.119	1.63 (0.84 to 3.19)
Peripartum haemorrhage	01/09/2007 to 31/03/2009¶	1 176 025	152 884	67 269	31	46.0 (31.3 to 65.4)	85 616	51	59.5 (44.3 to 78.3)	0.259	0.77 (0.48 to 1.23)
Failed intubation	01/04/2008 to 31/03/2010	1 504 593	195 597	86 062	2	2.3 (0.2 to 8.3)	109 535	5	4.5 (1.4 to 10.6)	0.476	0.51 (0.05 to 3.11)

*Proportion of maternities likely to have undergone previous CS (based on 13% calculated from the sepsis controls, 2012–2013, drawn from the general population).

†Proportion of maternities with previous CS likely to undergo VBAC (based on 44% calculated from UKOSS-uterine rupture controls as was done by Fitzpatrick *et al*).

‡Proportion of maternities with previous CS likely to undergo ERCS (based on 56% calculated from UKOSS-uterine rupture controls as was done by Fitzpatrick *et al*).

§For counts <5, Fisher's exact test was carried out instead of Pearson's χ^2^ test.

¶The total maternities for this 18-month study were calculated as: total maternities in 2008+half the maternities in 2009, as the number of maternities found for 2007 (from the same sources) did not appear to be in complete agreement with the 2005–2012 trend. Owing to this uncertainty in the 2007 numbers, the 2008 and 2009 maternities have been used here.

CS, caesarean section; ERCS, elective repeat caesarean section; VBAC, vaginal birth after caesarean.

### Statistical analyses

We calculated the incidence of each of the outcomes of interest with 95% CIs for the two exposure groups, VBAC and ERCS, using the denominators: total expected VBAC and ERCS maternities in a given year. The method of calculating proxy denominators was similar to that used by Fitzpatrick *et al*^8^ to report the incidence of uterine rupture in the VBAC and ERCS groups. Total maternities for the UK in the study period for each of the outcomes were calculated from the annually reported birth data for England and Wales,^18^ Scotland^19^ and Northern Ireland.^20^ From these, we calculated the estimated number of maternities likely to have undergone previous caesarean section, which was 13% of the total maternities, derived from a group of population-based controls comprised of women giving birth in the UK in 2012–2013. On the basis of the proportions observed in the control group of the UKOSS uterine rupture study,^8^ we further divided the maternities with previous caesarean section into women undergoing planned VBAC (44% of the total maternities with previous caesarean section) and women undergoing planned ERCS (56% of the total maternities with previous caesarean section), which gave the required proxy denominators.

In addition, we also tested whether the calculated rates in the exposure groups were significantly different from each other using χ^2^ test or Fisher's exact test. We estimated the risk ratios and 95% CIs to ascertain the relative risk of severe maternal morbidities in the planned VBAC group compared with the ERCS group. We also used descriptive statistics to compare the two exposure groups. In order to account for any differences in known and potential confounding factors, we conducted multivariable logistic regression analyses for the outcomes for which we had a control group: peripartum hysterectomy, sepsis and failed intubation. The multivariable logistic regression analysis results for uterine rupture have been published previously.^8^

In the sample for sepsis, 11 cases could not be classified into VBAC or ERCS due to missing information, and peripartum haemorrhage and hysterectomy each had one case with missing information (figure 1). We conducted a sensitivity analysis by calculating incidence rates assuming extreme scenarios and accordingly including the missing numbers under each of the two exposure groups.

## Results

A total of 83 confirmed cases of peripartum haemorrhage, 66 cases of hysterectomy, 49 cases of severe sepsis and 7 cases of failed tracheal intubation were included in the study (figure 1). The exposure groups, ERCS and planned VBAC, for each of the outcomes were not significantly different in terms of maternal age, body mass index (BMI), parity, history of previous pregnancy problems and socioeconomic status. The calculated incidence rates of the maternal complications were low and were not found to be significantly different between the two groups (table 2). The relative risk of the severe maternal morbidities was not different between the VBAC and ERCS groups (table 2).

The unadjusted ORs for the adverse outcomes, for which we had a control group, were not significantly different between the VBAC and ERCS groups (peripartum hysterectomy: unadjusted OR (uOR)=0.86, 95% CI 0.46 to 1.62; sepsis: uOR=0.51, 95% CI 0.24 to 1.07; failed intubation: uOR=0.36, 95% CI 0.02 to 5.11). The adjusted odds of peripartum hysterectomy (adjusted OR=0.92; 95% CI 0.45 to 1.91) and sepsis (adjusted OR=0.51; 95% CI 0.22 to 1.19) in the ERCS group were not significantly different from those of the VBAC group after controlling for current and previous pregnancy problems, number of previous caesarean sections, pre-existing medical problems, parity, smoking status, socioeconomic status, ethnic background, marital status, BMI and maternal age. The adjusted OR was not meaningful for failed intubation which had a total sample size of 15.

Sensitivity analysis for cases with missing information showed that although the rates changed slightly in the planned VBAC and ERCS groups for peripartum haemorrhage and hysterectomy, it did not result in a significant difference in the risk of the adverse outcome between the two exposure groups in either scenario. However, for severe sepsis, when all the 11 cases with missing information were included in the planned VBAC group, the rate in the VBAC group was found to be significantly higher than the rate in the ERCS group (p value for χ^2^ test=0.002). When these cases were included in the ERCS group, the rates of sepsis in the two exposure groups were equal (50 per 100 000 maternities).

## Discussion

This study, which used the UKOSS data and a nested retrospective cohort design, did not find a significant difference in the incidence and relative risk of adverse maternal outcomes between the VBAC and ERCS groups. However, the incidence rates of these outcomes were low.

### Strengths and limitations

The low incidence of severe maternal morbidities in the UK makes it difficult to compare the risks between the VBAC and ERCS groups. The UKOSS database of research data on rare and potentially life-threatening conditions in pregnancy provided a unique opportunity to estimate the risk of four adverse maternal outcomes between the two groups in a cost-effective manner. The method used to generate the exposure groups (planned VBAC and ERCS) could have misclassified some women who were planning ERCS, but went into spontaneous labour and were thus included in the VBAC group. However, we do not anticipate a large proportion of such women. Cases which could not be grouped into VBAC or ERCS due to missing information could have biased the study results, mainly for the sepsis group. We have thus reported the results of a sensitivity analysis. Further, including a proxy denominator calculated from a control population comprising women giving birth in 2012–2013 assumes that the rate of caesarean sections and proportions of VBAC and ERCS did not vary over the time periods between 2005–2006 and 2012–2013. Considering that population-level caesarean section rates for the UK per year were not available from any source, we employed this alternative method used in a previous study by Fitzpatrick *et al.*^8^ Although we accounted for known confounders for each outcome in the multivariable logistic regression analyses, the results cannot be interpreted with certainty due to the small sample sizes. While we excluded women diagnosed antenatally with placenta praevia/accreta/percreta, we did not have information on other potential absolute indications for ERCS which could bias the study results. A longer term study with a greater number of cases would improve the power and possibly show significant differences in the outcomes; however, this study intended to take advantage of existing secondary data, and the results could pave the way for further studies. Furthermore, the adverse outcomes presented in this study are immediate risks associated with the current pregnancy in the ERCS and VBAC groups, and we cannot comment on the risk of morbidity in future pregnancies.

While the higher risk of uterine rupture associated with planned VBAC is known,^4^ ^8^ ^21^ ^22^ studies from different parts of the world have reported variable relative and absolute risks of other maternal complications in the ERCS versus VBAC group. Similar to the findings of this study, a multicentre prospective cohort study in the USA,^4^ a Canadian study^11^ and a meta-analysis of the literature published between 2000 and 2007^22^ did not find any difference in the risk of hysterectomy between those who underwent a trial of labour and those who had an elective caesarean section. However, a decision-model analysis conducted by Paré *et al*^23^ suggested that the decision to undergo VBAC or ERCS among women with one prior caesarean section should be guided by the number of planned subsequent pregnancies. On the basis of an analysis of risk of hysterectomy, the authors suggested that ERCS should be the strategy of choice for women planning one additional pregnancy, but for women who desire two or more subsequent pregnancies, VBAC should be attempted to minimise morbidity associated with multiple caesarean sections.^23^

In contrast to our findings, a study in Australia found a 63% lower risk of peripartum haemorrhage in the planned ERCS group compared with the planned VBAC group,^6^ and the multicentre study from the USA demonstrated a higher odds of transfusion in the VBAC group compared with the ERCS group.^4^ However, a meta-analysis suggested a lower risk of peripartum haemorrhage in the VBAC group,^21^ and other studies did not show any difference.^11^ ^22^ A prospective cohort study of obese women using data collected through the UKOSS did not find any difference in anaesthetic complications between the ERCS and planned VBAC groups,^24^ but this finding cannot be generalised to non-obese women.

## Conclusion

While the risk of uterine rupture in the VBAC and ERCS groups is well understood, this national study did not demonstrate any other clear differences in the outcomes we examined. The absolute and relative risks of maternal complications were small in both groups, which is important information to help pregnant women in their decision-making process. Large epidemiological studies with a longer time period for data collection are required to assess whether the incidence of these rare outcomes would significantly differ between the VBAC and ERCS groups if a larger number of cases were to be examined. In the interim, this study contributes additional information to the process of individualised decision-making about the mode of delivery by women who have had a previous delivery by caesarean section, as recommended in current guidance.
